# Lessons from previous predictions of HIV/AIDS in the United States and Japan: epidemiologic models and policy formulation

**DOI:** 10.1186/1742-5573-4-3

**Published:** 2007-06-13

**Authors:** Hiroshi Nishiura

**Affiliations:** 1 Department of Medical Biometry, University of Tübingen, Westbahnhofstr. 55-D, Tübingen, D-72070, Germany; 2 Research Center for Tropical Infectious Diseases, Nagasaki University Institute of Tropical Medicine, Sakamoto 1-12-4, Nagasaki, 852-8523, Japan

## Abstract

This paper critically discusses two previous studies concerned with predictions of HIV/AIDS in the United States and Japan during the early 1990s. Although the study in the US applied a historical theory, assuming normal distribution for the epidemic curve, the underlying infection process was not taken into account. In the Japan case, the true HIV incidence was estimated using the coverage ratio of previously diagnosed/undiagnosed HIV infections among AIDS cases, the assumptions of which were not supported by a firm theoretical understanding. At least partly because of failure to account for underlying mechanisms of the disease and its transmission, both studies failed to yield appropriate predictions of the future AIDS incidence. Further, in the Japan case, the importance of consistent surveillance data was not sufficiently emphasized or openly discussed and, because of this, revision of the AIDS reporting system has made it difficult to determine the total number of AIDS cases and apply a backcalculation method. Other widely accepted approaches can also fail to provide perfect predictions. Nevertheless, wrong policy direction could arise if we ignore important assumptions, methods and input data required to answer specific questions. The present paper highlights the need for appropriate assessment of specific modeling purposes and explicit listing of essential information as well as possible solutions to aid relevant policy formulation.

## Background

Although numerous mathematical and statistical approaches have been proposed to predict the future course of infectious diseases, various assumptions are required to account for the intrinsic and extrinsic dynamics of disease spread [1-3], and consequently, detailed models require specialized knowledge. Thus, it is often the case that the majority of non-experts directly adopt the predictions or estimates as given, neglecting key assumptions and/or significant flaws. This paper critically discusses two previous studies concerned with predictions of HIV/AIDS in the United States and Japan. Through these case studies, which include relatively obvious technical problems related to the intrinsic assumptions, I examine how model-based suggestions are interpreted among non-experts and attempt to suggest potential solutions for general quantitative models relevant to policy making.

## Analysis

### Mathematical and statistical models of HIV/AIDS epidemiology

Before entering into discussion on the details of the case studies, two widely accepted models of HIV/AIDS epidemiology are briefly discussed. Whereas there is no single best method for AIDS prediction, these two approaches, both of which were initially proposed during the late 1980s, are widely employed [4-10].

The first approach is based on a mathematical model that explores the population dynamics of HIV/AIDS [1,11]. The model is constructed in a bottom-up fashion, facilitating understanding of the qualitative patterns of transmission dynamics. It should be noted that some studies employ this method to explore qualitative or analytical features only [2], offering ecological implications (e.g., regarding evolutionary biology [12]) based on the observed qualitative patterns. The pioneering model on HIV/AIDS [4,7] and technical reviews of this model are given elsewhere [13,14]. Quantitative application of this approach is typically represented by the estimations and predictions performed by the Joint UN Programme on HIV/AIDS (UNAIDS) mainly in developing countries [15] using observed HIV prevalence data (e.g., from antenatal clinics or military personnel). Although the model structure and assumptions vary depending on the specific purpose of the study, advantages of this approach include a firm understanding of the intrinsic dynamics (e.g., how the contact structure contributes to the spread of HIV) [16,17] and analysis of the potential impact of interventions on HIV/AIDS epidemiology [18].

The second model, referred to as backcalculation, is a more statistically motivated approach to estimating HIV prevalence [8-10]. Backcalculation uses the statistical distribution of incubation period as key information, and is frequently applied to HIV/AIDS in industrialized countries where the previous AIDS incidence can be assumed to be confidently diagnosed and reported. The epidemic curve for HIV is reconstructed using AIDS incidence and the incubation period, enabling estimation of HIV prevalence and short-term projections of AIDS incidence. Since this paper mainly deals with HIV/AIDS in industrialized countries, the simplest form of this estimation method is given in Additional file 1, with technical details available elsewhere [19-21].

Neither the US nor Japan case studies used the above methods, and further, whereas the above two approaches account for underlying mechanisms of the disease, the two case studies focused almost entirely on extrapolation of the epidemic curve.

### The United States case

**Figure 1:**
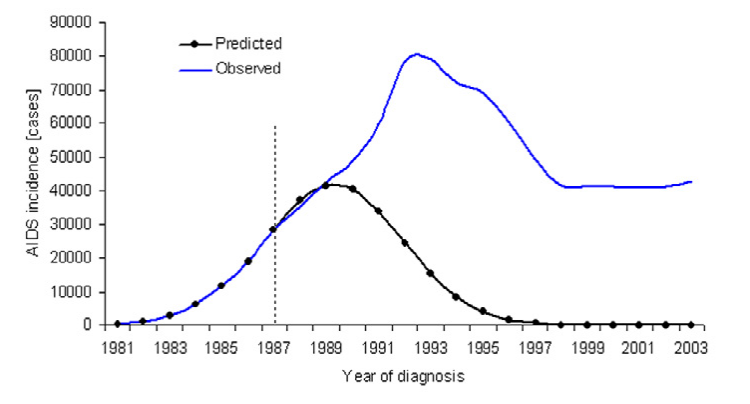
**Observed and predicted AIDS incidence in the United States from 1981–2003**. Data source: refs. [26,27]. Details of the underlying assumptions employed are described in the Additional file. The prediction was obtained using the data up to the dashed line; i.e., from 1981–7. The constant second ratio of AIDS incidence was 0.8647, as adopted in ref. [22].

The approach employed in the US case study assumed a normally distributed epidemic curve [22]. The underlying theory, referred to as Farr's law [23], was used in the late 1980s with direct application of the concept to the annual incidence of AIDS in the US [24,25]. Fig.1 shows the incidence of adult AIDS cases in the US by year of diagnosis from 1981–2003 [26,27] along with the predictions obtained in the above case study based on Farr's law [22]. Since the data include all routes of transmission, and because of the revised definition of diagnostic criteria and corrected number of cases, the annual incidence in Fig. 1 is slightly higher than in the original study [22], while maintaining similar trends and prediction results. Technical details are given in Additional file 1.

### The Japan case

As the Japan case study, the series of studies by the Working Group on prediction of HIV-infected cases and AIDS patients, organized by the Ministry of Health, Labor and Welfare (MHLW), Japan, are discussed [28-31]. From the very first study [28], the ratio of previously reported AIDS cases to unreported cases was used as key information, assuming that it was equivalent to the "coverage rate" of reported HIV infection (note: precisely, the 'ratio', not 'rate', of reported to unreported AIDS was used). In other words, it was assumed that the true HIV incidence could be estimated using the number of reported HIV infections with the coverage ratio directly obtained from the ratio of previously diagnosed (reported) to undiagnosed (unreported) AIDS cases. Thus, the following relationship was assumed:

HdHu=AdAu
 MathType@MTEF@5@5@+=feaafiart1ev1aaatCvAUfKttLearuWrP9MDH5MBPbIqV92AaeXatLxBI9gBaebbnrfifHhDYfgasaacH8akY=wiFfYdH8Gipec8Eeeu0xXdbba9frFj0=OqFfea0dXdd9vqai=hGuQ8kuc9pgc9s8qqaq=dirpe0xb9q8qiLsFr0=vr0=vr0dc8meaabaqaciaacaGaaeqabaqabeGadaaakeaadaWcaaqaaiabdIeainaaBaaaleaacqWGKbazaeqaaaGcbaGaemisaG0aaSbaaSqaaiabdwha1bqabaaaaOGaeyypa0ZaaSaaaeaacqWGbbqqdaWgaaWcbaGaemizaqgabeaaaOqaaiabdgeabnaaBaaaleaacqWG1bqDaeqaaaaaaaa@3870@

where *H*_*u*_, *H*_*d*_, *A*_*u*_, and *A*_*d *_are the cumulative number of unreported and reported HIV infections and unreported and reported AIDS cases, respectively. The ratio has been revised several times, but the most recent studies adopt an estimate of 1/5.1 (reported/unreported), because approximately 19.6% of the AIDS cases reported up until the mid-1990s were previously diagnosed as being infected with HIV [30,32].

The ratio should be implicitly assumed to be independent of time. Using the estimated HIV prevalence based on this ratio, it is assumed that HIV-infected individuals develop AIDS according to a fixed incubation period distribution that follows Weibull distribution [28-30]. Based on the above assumptions, the future number of HIV infections as well as the future AIDS incidence was obtained, assuming that a constant increase in HIV infections would occur [28,29]. That is, a linear trend was adopted for the reports of HIV infection.

### Failure to reflect the reality

As shown in Fig. 1, the AIDS projection obtained in the US assuming a normal distribution for the temporal pattern of AIDS incidence failed to reflect the reality. Considering the shape of the epidemic curve and that the actual peak of AIDS incidence was seen later than predicted, it seems that the basic assumptions of this projection were inappropriate, resulting in serious underestimation. The inaccuracy was suggested immediately after publication of the original study [33]. Their argument was based on an HIV seroprevalence survey conducted in the late 1980s in which the estimated prevalence of HIV was 5-fold larger than the predicted cumulative number of future AIDS cases obtained from normal distribution [34].

The original study did not take into account the reason behind the assumption of a normal curve [22,35]. Non-normal distribution originates from the characteristics of the nationwide data under the influence of immigration of infected individuals, a right-skewed incubation period distribution of HIV infection and further modification as a result of antiretroviral therapy [36]. However, a more significant flaw concerns the reason why a symmetric bell-shaped curve could be assumed for the epidemic curve. Provided that the assumption of a normal distribution had been empirically confirmed, the technical problems related to HIV/AIDS-specific intrinsic dynamics might have been justifiable during the 1980s. The potential reason for assuming a normal epidemic curve is given in Additional file 1, but was not appropriately understood in the US study.

**Figure 2:**
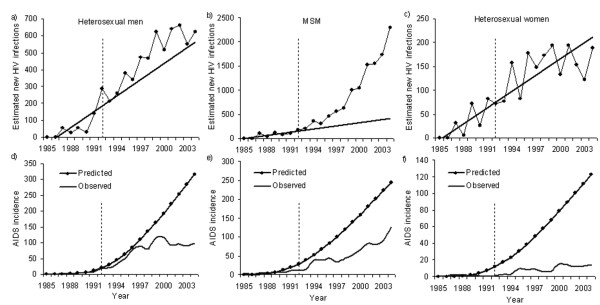
**Observed and predicted numbers of HIV infections and AIDS diagnoses in Japan from 1985–2004**. Data source: ref. [44]. The top three panels show the estimated true HIV incidence (a-c) and the bottom show AIDS incidence (d-f). The routes of transmission are heterosexual men (a and d), men who have sex with men (MSM; b and e), and heterosexual women (c and f). The prediction was obtained using the data up to the dashed line; i.e., from 1985–92. The straight lines in a-c represent the predicted true HIV incidence based on the assumption of linear growth. The coverage ratio was 1/5.1 [30,32]. The shape and scale parameters for the Weibull distribution used to describe the incubation period were 2.286 and 10.0, respectively, as adopted in ref. [28].

Figs. 2a–c show the predicted true HIV incidences based on the above mentioned assumptions for three major routes of transmission in Japan. The annual reported number of HIV infections from 1985–1992 and a coverage ratio of 1/5.1 were used for these predictions. The observed true HIV incidences in the figure were obtained by multiplying the inverse of the coverage ratio (5.1) by the reported number of HIV infections in each year. Figs. 2d–f compare the observed and predicted AIDS incidences based on the HIV prevalence estimates shown in Figs. 2a–c. For all three routes of transmission, the predictions were clearly overestimated compared to the actual AIDS incidence. Although later work applied a dynamical system to exploration of the potential impacts of interventions [31], the data source for this model was also based on estimates of true HIV incidence using the coverage ratio. There were two obvious technical flaws: (1) a linear growth trend for HIV was adopted without extensive validation, and (2) the coverage ratio did not approximate the ratio of previously reported to unreported HIV infections. Analytical discussion of the latter point is highlighted in Additional file 1.

### The impact of these failures on public health

A number of professionals immediately and sharply criticized the US projection [22,33,36,37]. Issues related to the extrapolation of epidemic curves, the functional form of which significantly influences the predictions, are explained in detail elsewhere [35]. In addition to these immediate criticisms and a technical review of HIV/AIDS predictions [33-37], at least another six articles referenced the US study [38-43]. Although technical criticisms regarding the obvious flaws have been noted, and even though care seems to have been taken to appropriately interpret the prediction, the US study was simply described as ''a more conservative estimate'' in a general policy publication reviewing various predictions in the US [38].

Some publications, which did not follow the widely discussed theoretical concepts, investigated the original study as an accepted theory or extended the discussion concerning predictability. Shortly after publication of the original study and more recently [39,40], the above analogy was referenced as formal theoretical evidence explaining the mechanistic process of the bell-shaped pattern of AIDS incidence. Similarly, the original study has been referenced several times as a theory explaining the declining pattern of HIV infections [41,42]. Moreover, a lack of careful interpretation among non-experts has also led to another epidemiologic flaw. A later study suggested the usefulness of a simple linear regression (rather than Farr's law), claiming that the regression left less than 10% residual variance in incidence [43]. Even though criticisms were made among specialists, and thus, the original study had little impact on public health in the US, non-experts often remain unaware of the detailed mechanism of disease spread (i.e., the reason why the original study did not reflect the reality).

In Japan, the Committee of AIDS Trends, formed by MHLW, expressed their standpoint with respect to interpretation of such estimations as follows [44]: "Because there are various methods that could be used for estimations, the Committee considers it difficult to identify the single best method. Thus, it is not appropriate to present estimated numbers using a specific method. Rather, we suggest that the estimates of individual researchers should be employed while referring to the underlying assumptions or hypothesis of the methods used" (author's translation). Studies based on the presented Japan case study [28-31] as well as on different assumptions [45] were given as references in this report. Unfortunately, the latter study was similarly not based on widely accepted intrinsic assumptions [45]. Despite the above statement, the findings of the Japan studies [28-31] are most frequently adopted as official estimates [46,47], because only these studies consecutively provided predictions for Japan.

The Japan case study also indirectly influenced the AIDS notification method. Prior to revision of the surveillance system in 1999, HIV/AIDS notifications among Japanese nationals were classified into one of three categories: (1) HIV infection, (2) AIDS without a previous diagnosis of HIV infection (= 'First Report'), and (3) AIDS with a previous HIV diagnosis (= 'Second Report'). Since implementation of the Law Concerning the Prevention of Infectious Diseases and Medical Care in 1999, replacing the previous law for HIV/AIDS in Japan, it has become difficult to link individuals initially categorized as (1) and later as (3) due to the altered administrative system [48]. Moreover, the Second Report was often delayed because many physicians did not feel particularly obliged to report AIDS cases that had already been documented as HIV infected [32]. Thus, owing to the suggestion that 'Second Reports are confusing', they became non-obligatory after 1999 and, since then, individuals categorized as such have not been included in the total number of AIDS cases. This made it impossible not only to assess the robustness of the assumption using the coverage ratio but also to perform estimations using backcalculation, which essentially requires the total number of AIDS cases [8-10]. If the importance of the total number of AIDS cases had been sufficiently highlighted and openly discussed in detail, revision of the reporting system would not have been necessary.

### Policy-making and the modeling framework

The two case studies discussed here employed rather different approaches compared to widely accepted methods. In the US, experts in relevant fields criticized the original study and the flawed prediction has become relatively well known, at least among specialists. Although there were few public health impacts (including the data collection method), a few non-experts, who seem not to recognize the importance of intrinsic dynamics, continue to reference and extend the original theory. Moreover, the predictions obtained based on the original Japan case study have been largely adopted as official, and unfortunately, only one study has so far criticized the technical flaw [49]. The following three points summarize what should be addressed. Many of the criticisms below are also common concerns for widely accepted models.

#### a) Common pitfalls in model-building

It should be accentuated that intrinsic dynamics are one of the most important factors offering valid and sound answers to the specific question addressed by the model (e.g., AIDS prediction in the above case studies) [50]. Transmission dynamics should be explored because observations of different individuals cannot be considered independently when studying infectious diseases. Although many widely accepted models have not overcome uncertainty or achieved precise predictions, their impact on our understanding of the epidemiologic process of HIV/AIDS have been discussed elsewhere [11,51]. The above two case studies were most likely unaware of the importance of intrinsic dynamics. This technical point should be further consulted elsewhere [11,50,52,53].

#### b) General assessment of prediction

It should be emphasized that even if a model has good predictability it does not necessarily mean it's a good model for prediction. It should also be noted that good models are essentially accompanied by valid basic assumptions of the spread of disease and pay close attention to the input parameters; the widely accepted approaches take particular care to heed this point. As in the above two case studies, the majority of flawed predictions employ simple extrapolation of an epidemic curve, the most uncertain component of modeling, frequently focusing on the predicted value itself or simple residual variance alone.

#### c) Assessment of modeling studies for policy making

One of the biggest challenges facing modelers and policy makers is how to communicate and share these points effectively [53]. Many researchers, including epidemiologists in other fields, do not sufficiently understand the basic model structures as well as key assumptions. Further, for the general reader, there are no general rules for evaluating original studies or critically assessing modeling studies. This is the case not only in the presented extreme cases but also with other common approaches frequently employed. Further, it is not uncommon for the scope and key assumptions to be insufficiently shared with policy makers before entering into detailed discussion of their predictability.

### Potential solutions

The criticisms presented here do not blame the authors of the presented case studies, but rather attempt to give general suggestions for improvement. As a possible remedy for modeling frameworks, to make them truly useful for policy making, I suggest the following potential solutions. These apply not only to the above discussed extreme cases in the US and Japan but also to most quantitative models.

#### a) Purposes of modeling

The model structure depends on investigation of a specific question. Even if based on valid assumptions, it must be remembered that models may not provide any insights other than their main purpose. Thus, what can and cannot be learned from each study must be explicitly documented for the reader to assess. If more than two quantitative approaches are applied to the same data, the purposes and information obtained could be different. It is not uncommon for various approaches to be applied to the same data, with the generated findings enhancing our understanding of the validity of the assumptions made. Thus, where possible, various approaches that differ from each other in their purposes should be applied to the same data.

#### b) List of assumptions, limitations and parameters

As discussed above, the validity of estimations and/or predictions largely depends on the assumptions made. Thus, it would be very useful if all publications documented the assumptions, limitations and input parameters in an explicit manner (for example, see [54,55]). Although such documentation is a widely accepted practice in current modeling papers, it might be further useful if the information was systematically listed. Moreover, it would be worth documenting the role of each parameter employed, the source of the parameter estimates, and the geographic representativeness. For policy makers, it might be useful to check if modeling studies follow such rules of listing.

#### c) Assumptions employed to answer specific questions

Most modeling frameworks are accompanied by a certain number of unrealistic assumptions. Despite efforts to make a model as realistic as possible, it is often the case that the data is limited, e.g., as a result of future improvements in treatments or behavioral changes. It should be noted, however, that incorporation of numerous details is not necessarily a remedy for inappropriate description of the reality. Depending on the question being addressed, an accurate yet simplistic model may be desirable; a simple model often better reflecting the reality than complex simulations [11]. In line with this, non-experts are advised to assess two points regarding such studies: (1) whether the model successfully answers the addressed question in a simple fashion, and (2) whether the assumptions made are sound and sufficiently realistic.

#### d) Policy formulation

Modelers should fully understand the intrinsic dynamics of the disease and/or work closely with specialists who can offer detailed disease information. Since it is not uncommon for the findings of a model to be misinterpreted (as in the above case studies), it is crucial that modelers, policy makers, and clinicians work together when creating and utilizing such models. Through such practice, modeling experts should share their knowledge in assessing the validity of a model and translating the implications for policy makers. Further, if public health officials are unable to assess the findings of model-based predictions, modelers should be on hand for consultation. These experts should also employ their knowledge to disease monitoring systems.

## Conclusion

The present study critically discussed two predictions of AIDS incidence with the aim of understanding how such estimates are interpreted by the general public and exploring possible improvements in policy making. It should be emphasized that ignoring how a disease spreads, and related assumptions, as well as neglecting key input information could lead to wrong policy direction. The need for appropriate assessment of specific modeling purposes, and explicit listing of assumptions, limitations and parameter inputs as well as possible solutions to aid relevant policy formulation was therefore highlighted.

## Competing interests

The author(s) declare that they have no competing interests.

## Authors' contributions

HN carried out paper reviews, proposed the study, performed mathematical analyses and drafted the manuscript. The author has read and approved the final manuscript.

## Supplementary Material

Additional File 1 **Additional methods**. Mathematical details are provided in pdf format. Click here for file
