# Supplementary material for: Lessons from previous predictions of HIV/AIDS in the United States and Japan: epidemiologic models and policy formulation
Source: Epidemiol Perspect Innov. 2007 Jun 13;4:3. doi: 10.1186/1742-5573-4-3 (PMC1906780; doi:10.1186/1742-5573-4-3)
Supplement: Additional File 1 — Additional methods. Mathematical details are provided in pdf format. [file 1742-5573-4-3-S1.pdf]

Additional file

Supplement to: Nishiura H: **Lessons from previous predictions of HIV/AIDS in the United States and Japan: Epidemiologic model and policy formulation**. In: *Epidemiologic Perspectives and Innovations*.

Backcalculation: Reconstruction of the HIV epidemic

Backcalculation is a useful technique for estimating HIV prevalence and obtaining short-term projections of AIDS incidence based on previous AIDS incidence data [1-3]. Whereas the number of AIDS cases is thought to be relatively accurately documented in industrialized countries, asymptomatic HIV infections are seldom noticed unless the infected individual undertakes a voluntary blood test or develops the disease. The long incubation period of HIV infection enables assessment of the extent of the epidemic during its course. Backcalculation uses AIDS incidence data at time t, $a(t)$, and the incubation period distribution at time τ after infection, $\omega(\tau)$, to reconstruct the number of HIV infections with time. Assuming that documentation of diagnosed AIDS cases is not significantly delayed, and assuming the impact of antiretroviral therapy on the length of the incubation period is negligible in the simplest setting, the fundamental relationship is given by the following convolution equation:

$$a(t) = \int_0^t h(u)\omega(t-u)du, \quad (1)$$

where $h(u)$ is the number of HIV infections at time u. The basic idea of backcalculation is to obtain $h(u)$ using $a(t)$ and $\omega(\tau\text{-}u)$. Here, to ease understanding of the deconvolution procedure, eqn. (1) is considered in discrete time [4,5]. Since

surveillance-based data of AIDS incidence is obtained for a certain interval, t (e.g., every 2 or 3 months), the following equation is obtained:

$$a_t = \sum_{u=1}^{t} h_u \omega_{t-u}. \quad (2)$$

Assuming that h_t is generated by a nonhomogeneous Poisson process, a_t is an independent Poisson variate. Thus, the likelihood, which is needed to estimate HIV infections and the parameters of incubation period distribution, is proportional to:

$$\prod_{t=1}^{T} \left(\sum_{u=1}^{t} h_u \omega_{t-u} \right)^{r_t} \exp\left(-\sum_{u=1}^{t} h_u \omega_{t-u} \right), \quad (3)$$

where r_t is the observed number of AIDS cases at time t and T is the most recent time of observation. The shape of the curve of HIV infections, h_t, is usually modeled parametrically or non-parametrically. The main sources of uncertainty arise from uncertainties in the incubation period distribution, the shape of the HIV infection curve, and AIDS incidence data [6]. Short-term predictions are obtained based on estimated numbers of HIV infected individuals who have not yet developed AIDS. However, it should be noted that backcalculation such as this provides no information about future infection rates and little information about recent infection rates [7]. Further details of the backcalculation method are described elsewhere [8-10].

The prediction method employed in the United States case study

In the United States case study, future predictions of AIDS incidence based on the annual number of AIDS diagnoses from 1981-7 were estimated using the second ratio of incidence, which reflects the annual incidence of AIDS (see Table S1). That is, empirically, assuming that the second ratio of AIDS incidence is constant, and thus, the epidemic curve suffices to fit a normal curve, the first ratio and future AIDS

incidence can subsequently be obtained arithmetically [11]. In the original study [12], the fixed second ratio was determined as 0.8647 using the mean ratio from 1985-7; Table 1 also follows this estimate.

Farr's law was formalized in detail by John Brownlee (1868-1927) [13] who, based on the observational notes of Farr on the temporal pattern of smallpox death, showed that epidemics in general tend to follow a symmetric bell-shaped curve that can be approximated by normal distribution [14,15]. The major aim of Brownlee in extending this theory was to further investigate the time-series decline of transmission potential (i.e., infectiousness) during the course of an epidemic, which he failed to do (excellent historical reviews of Brownlee's efforts are given elsewhere [16,17]). Assuming that the second ratio of AIDS incidence, which reflects the annual incidence, is constant over time, the following fundamental equation is obtained:

$$\frac{a_1 / a_2}{a_3 / a_4} = \frac{a_2 / a_3}{a_4 / a_5} = C < 1, \quad (4)$$

where a_t and C are the AIDS incidence at time-interval t and the assumed constant second ratio, respectively. Brownlee found that eqn. (4) can also be described by the second differences of the following logarithms:

$$(\ln a_1 - \ln a_2) - (\ln a_3 - \ln a_4) = \ln C. \quad (5)$$

When $\ln a = A$ the above equation can be expressed by:

$$\frac{d^2 A}{dt^2} = \ln C. \quad (6)$$

Thus, the solution to eqn. (6) can be obtained from the integral:

$$a(t) = \exp(-At^2 + Bt + D). \quad (7)$$

In other words, a negative second-degree exponential function, which describes a type of normal distribution, is obtained [13,16,17].

A theoretical flaw of the AIDS projection in the United States

The most significant flaw lies in the underlying theory. Provided that the assumption of a normal distribution had been empirically confirmed for other infectious diseases [18] and that functions similar to those given in eqn. (7) are frequently assumed for the infection rate of HIV even in recent backcalculation methods, the technical problems in HIV/AIDS-specific intrinsic dynamics might have been justifiable during the 1980s. The flaw is concerned with the reason why a symmetric bell-shaped curve could be assumed for the epidemic curve. Farr and Brownlee could not clarify the mechanism behind the normal curve according to the underlying infection process [11,15,16], and consequently, Bregman and Langmuir adopted an assumption not clarified in the explicit bottom-up fashion [12].

If a symmetric bell-shaped curve was needed, a susceptible (S)-infectious (I)-removed (R) model (an SIR model), one of the most widely used assumptions based on the work by Kermack and McKendrick [19], could have been used based on the mass action principle firstly suggested by Hamer [20,21]. The basic differential equations describing the population dynamics of an epidemic are given by:

$$\frac{dS}{dt} = -\beta SI,$$
$$\frac{dI}{dt} = \beta SI - \gamma I, \qquad (8)$$
$$\frac{dR}{dt} = \gamma I,$$

where β and γ are the infection and removal rates, respectively. Assuming the epidemiology of HIV/AIDS, $I(t)$ and $R(t)$ are the number of HIV infections and AIDS cases at time t (note: strictly speaking, the simple mass action principle is likely inappropriate for HIV/AIDS). Thus, the epidemic curve, i.e., AIDS incidence, is given

by the solution of $dR(t)/dt$. Here, to clearly show the analytical results, the incubation period of HIV is assumed to follow an exponential distribution with the mean γ^{-1} and β assumed to be independent of time. Replacing $I(t)$ in the first subequation with the third, we have:

$$\frac{1}{S}\frac{dS}{dt} = -\frac{\beta}{\gamma}\frac{dR}{dt}. \qquad (9)$$

Integrating eqn. (9) from 0 to t yields:

$$S(t) = S(0)\exp\left\{-\frac{\beta}{\gamma}\big(R(t)-R(0)\big)\right\}, \qquad (10)$$

where $R(0) = 0$. Assuming that the total number of individuals in the population is constant $N = S(t) + I(t) + R(t)$ for any t, the third subequation of eqns. (8) can be written as:

$$\frac{dR}{dt} = \gamma\left\{N - R(t) - S(0)\exp\left(-\frac{\beta}{\gamma}R(t)\right)\right\}. \qquad (11)$$

Approximation of eqn. (11) by Taylor series expansion yields:

$$\frac{dR}{dt} = \gamma\left\{N - S(0) + \left(\frac{\beta S(0)}{\gamma} - 1\right)R(t) - \frac{\beta^2 S(0) R(t)^2}{2\gamma^2}\right\}. \qquad (12)$$

This can be solved by standard methods [22] yielding:

$$R(t) = \frac{\gamma^2}{\beta^2 S(0)}\left\{\frac{\beta S(0)}{\gamma} - 1 + \alpha \tanh\left(\frac{1}{2}\alpha\gamma t - \phi\right)\right\}, \qquad (13)$$

where $\alpha = \sqrt{\left(\frac{\beta S(0)}{\gamma} - 1\right)^2 + \frac{2\beta^2 S(0) I(0)}{\gamma^2}}$ and $\phi = \tanh^{-1}\frac{1}{\alpha}\left(\frac{\beta S(0)}{\gamma} - 1\right)$.

Therefore, the epidemic curve is given by:

$$\frac{dR}{dt} = \frac{\alpha^2\gamma^3}{2\beta^2 S(0)}\operatorname{sech}^2\left(\frac{1}{2}\alpha\gamma t - \phi\right), \qquad (14)$$

generating a symmetrical bell-shaped epidemic curve. In this way, the epidemic curve obtained using the Kermack and McKendrick model results in a symmetric shape,

reflecting the decline of susceptible individuals (when β is constant over time). This indicates that the underlying epidemiologic process of the epidemic curves described by normal family (i.e., those which can be generalized with a type of normal distribution) partly originates from these non-linear dynamics. Bregman and Langmuir's study on the projection of HIV/AIDS in the United States [12], which simply applied the original historical theory to the data, did not validate the underlying intrinsic transmission dynamics using firm knowledge. In other words, they did not take into account the reason behind the assumption of a normal curve.

A technical flaw of the fixed coverage ratio employed in the Japan case study

Here I analytically examine the validity of applying the fixed coverage ratio to estimates of the true HIV incidence in Japan. Fig. S1 shows a schematic illustration of the four compartments required for this analysis. New HIV infections join compartment h_u, undiagnosed HIV infections, as a function of time t, $\eta(t)$. Undiagnosed HIV-infected individuals develop AIDS at a rate of γ_1 and are diagnosed at a rate of α (and enter compartment h_d, diagnosed HIV infections). Diagnosed HIV individuals develop AIDS at a rate of γ_2. This simple model is described by the following differential equations:

$$
\begin{aligned}
\frac{dh_u}{dt} &= \eta(t) - (\alpha + \gamma_1)h_u, \\
\frac{dh_d}{dt} &= \alpha h_u - \gamma_2 h_d, \\
\frac{da_u}{dt} &= \gamma_1 h_u, \\
\frac{da_d}{dt} &= \gamma_2 h_d,
\end{aligned}
\qquad (15)
$$

where γ_1 and γ_2 are unrealistically assumed to follow exponential distributions, a simplification that allows us to find the analytical solution. In the following analysis, the disease age (i.e., time since HIV infection) is ignored, and diagnosed and undiagnosed individuals are assumed to develop AIDS at the same rate, $\gamma (= \gamma_1 = \gamma_2)$. However, it should be noted that it is preferable to take into account the disease age (known as the *d*-state [23]) with slowly progressing diseases such as HIV/AIDS; analysis taking into account disease age is given elsewhere [24]. The rate of diagnosis, α, is assumed to be sufficiently small compared to γ and independent of time so that the coverage ratio in the original study in relation to time could be analytically obtained. Reporting delays and delayed onset of disease with antiretroviral therapy is also ignored for simplicity. In order to model the increase in new HIV infections in the simplest theoretical form, and based on the results of discrete backcalculation numerically developed for the purpose of introducing the concept into Japan [25], the time-dependent function of new HIV infections, $\eta(t)$, was assumed to follow a simple exponential function:

$$\eta(t) = \eta_0 \exp(\rho t). \qquad (16)$$

Then, the analytical solution of eqn. (15) is given by the following:

$$\begin{aligned}
h_u(t) &= \frac{\eta_0}{\rho+\alpha+\gamma}\left\{\exp(\rho t) - \exp(-(\alpha+\gamma)t)\right\},\\
h_d(t) &= \frac{\alpha\eta_0}{\rho+\alpha+\gamma}\left\{\frac{\exp(\rho t)-\exp(-\gamma t)}{\rho+\gamma} - \frac{\exp(-\gamma t)-\exp(-(\alpha+\gamma)t)}{\alpha}\right\},\\
a_u(t) &= \frac{\gamma\eta_0}{\rho+\alpha+\gamma}\left\{\frac{\exp(\rho t)-1}{\rho} - \frac{1-\exp(-(\alpha+\gamma)t)}{\alpha+\gamma}\right\},\\
a_d(t) &= \frac{\alpha\gamma\eta_0}{\rho+\alpha+\gamma}\left[\frac{1}{\rho+\gamma}\left\{\frac{\exp(\rho t)-1}{\rho} - \frac{1-\exp(-\gamma t)}{\gamma}\right\} - \frac{1}{\alpha}\left\{\frac{1-\exp(-\gamma t)}{\gamma} - \frac{1-\exp(-(\alpha+\gamma)t)}{\alpha+\gamma}\right\}\right]
\end{aligned} \qquad (17)$$

Thus, the ratio of HIV and AIDS diagnoses at time t is given as follows:

$$\frac{a_d(t)}{a_u(t)} = \frac{\dfrac{\alpha}{\rho+\gamma}\left\{\dfrac{\exp(\rho t)-1}{\rho} - \dfrac{1-\exp(-\gamma t)}{\gamma}\right\} - \left\{\dfrac{1-\exp(-\gamma t)}{\gamma} - \dfrac{1-\exp(-(\alpha+\gamma)t)}{\alpha+\gamma}\right\}}{\left\{\dfrac{\exp(\rho t)-1}{\rho} - \dfrac{1-\exp(-(\alpha+\gamma)t)}{\alpha+\gamma}\right\}} \qquad (18)$$

$$\frac{h_d(t)+a_d(t)}{h_u(t)+a_u(t)} = \frac{\alpha\left\{\frac{\exp(\rho t)-\exp(-\gamma t)}{\rho+\gamma} - \frac{\exp(-\gamma t)-\exp(-(\alpha+\gamma)t)}{\alpha}\right\} + \alpha\gamma\left[\frac{1}{\rho+\gamma}\left\{\frac{\exp(\rho t)-1}{\rho} - \frac{1-\exp(-\gamma t)}{\gamma}\right\} - \frac{1}{\alpha}\left\{\frac{1-\exp(-\gamma t)}{\gamma} - \frac{1-\exp(-(\alpha+\gamma)t)}{\alpha+\gamma}\right\}\right]}{\left\{\exp(\rho t)-\exp(-(\alpha+\gamma)t)\right\} + \gamma\left\{\frac{\exp(\rho t)-1}{\rho} - \frac{1-\exp(-(\alpha+\gamma)t)}{\alpha+\gamma}\right\}}$$

Here, the coverage ratio adopted in the above Japanese studies is given by $a_d(t)/a_u(t)$, which was believed to approximate $\{h_d(t)+ a_d(t)\}/ \{h_u(t)+ a_u(t)\}$; however, they were shown to be analytically unequal (i.e., $H_d/H_u \neq A_d/A_u$). Fig. S2a shows the time-dependent variation in the coverage ratio (based on previous diagnoses of AIDS cases) and the ratio of diagnosed HIV infections (including those who developed AIDS) assuming a constant diagnosis rate and exponential increase in HIV with time. Both ratios varied widely according to time (i.e., they were not independent of time) and, reflecting this, the coverage ratio in actual observations has been floated and revised several times [26-30]. The relationship between H_d/H_u and A_d/A_u is examined by various rates of diagnosis at time t = 7 [years] in Fig. S2b. It should be noted that even a slight difference in these ratios could result in significantly biased estimates of the true HIV incidence (or prevalence), since the estimate applies to the nationwide level. Moreover, previous mathematical analysis taking into account the *d*-state has claimed that the two ratios are not even approximately equal [24]. Thus, the coverage ratio used above should not be used to estimate the true HIV incidence and prevalence.

References

1. Brookmeyer R, Gail MH: **Minimum size of the acquired immunodeficiency syndrome (AIDS) epidemic in the United States**. *Lancet*. 1986, **2**: 1320-1322.
2. Brookmeyer R, Gail MH: **A method for obtaining short-term projections and lower bounds on the size of the AIDS epidemic**. *J. Am. Stat. Assoc.* 1988, **83**: 301-308.
3. Gail MH, Brookmeyer R: **Methods for projecting course of acquired immunodeficiency syndrome epidemic**. *J. Natl. Cancer. Inst.* 1988, **80**: 900-911.
4. Day NE, Gore SM, McGee MA, South M: **Predictions of the AIDS epidemic in the U.K.: the use of the back projection method**. *Philos. Trans. R. Soc. Lond. B. Biol. Sci.* 1989, **325**: 123-134.
5. Becker N: **Part 5: Data analysis: Estimation and prediction. Statistical challenges of epidemic data**. In *Epidemic Models: Their structure and relation to data*. Edited by Mollison D. Cambridge: Cambridge University Press; 1995:339-349.
6. Rosenberg PS, Gail MH: **Uncertainty in estimates of HIV prevalence derived by backcalculation**. *Ann. Epidemiol.* 1990, **1**: 105-115.
7. Ferguson NM, Donnelly CA, Woolhouse ME, Anderson RM: **The epidemiology of BSE in cattle herds in Great Britain. II. Model construction and analysis of transmission dynamics**. *Philos. Trans. R. Soc. Lond. B. Biol. Sci.* 1997, **352**: 803-838.

8. Brookmeyer R, Gail MH: *AIDS Epidemiology: A Quantitative Approach (Monographs in Epidemiology and Biostatistics)*. New York: Oxford University Press; 1994.
9. Jewell NP, Dietz K, Farewell VT: *AIDS Epidemiology: Methodological Issues*. Berlin: Birkhäuser; 1992.
10. Colton T, Johnson T, Machin D (Eds): **Proceedings of the Conference on Quantitative Methods for Studying AIDS**, held in Blaubeuren, Germany, June 14-18, 1993. In *Stat. Med.* 1994, **13**: 1899-2188.
11. Farr W: **Progress of epidemics**. In *Second Report of the Registrar General of England and Wales*. London: His majesty's Stationery Office; 1840:16-20, 91-98.
12. Bregman DJ, Langmuir AD: **Farr's law applied to AIDS projections**. *JAMA.* 1990, **263**: 1522-1525.
13. Brownlee J: **Statistical studies in immunity: the theory of an epidemic**. *Philos. R. Soc. Edinburgh.* 1906, **26**: 484-521.
14. Brownlee J: **On the curve of the epidemic**. *Br. Med. J.* 1915, **1**: 799-800.
15. Brownlee J: **Historical note on Farr's theory of the epidemic**. *Br. Med. J.* 1915, **2**: 250-252.
16. Fine PEM: **John Brownlee and the measurement of infectiousness: An historical study in epidemic theory**. *J. R. Stat. Soc. Ser. A.* 1979, **142**: 347-367.
17. Serfling RE: **Historical review of epidemic theory**. *Hum. Biol.* 1952, **24**: 145-166.
18. Wilson EB: **The logistic or autocatalytic grid.** *Proc. Natl. Acad. Sci.* 1925, **11**: 452-456.

19. Kermack WO, McKendrick AG: **Contributions to the mathematical theory of epidemics - I.** *Proc. R. Soc. Ser. A.* 1927, **115**: 700-721 (reprinted in *Bull. Math. Biol.* 1991, **53**: 33-55).
20. Hamer WH: **Epidemic disease in England: The evidence of variability and of persistency of type (Milroy Lectures III).** *Lancet.* 1906, **I**: 733-739.
21. Heesterbeek JAP: **The law of mass-action in epidemiology: a historical perspective**. In *Ecological Paradigms Lost: Routes of theory change*. Edited by Cuddington K and Besiner B. New York: Academic Press; 2005:81-105.
22. Bailey NTJ: **General epidemics**. In *The mathematical theory of infectious diseases and its applications (2nd ed)*. London: Griffin; 1975:81-132.
23. Diekmann O, Heesterbeek JAP: *Mathematical Epidemiology of Infectious Diseases: Model Building, Analysis and Interpretation.* New York: Wiley Series in Mathematical and Computational Biology; 2000.
24. Inaba H: **On trends of AIDS and an estimate for the number of HIV infecteds in Japan.** *J. Popul. Problems.* 1995, **50**: 31-44 (in Japanese with English abstract. Available at: http://www.ipss.go.jp/syoushika/bunken/DATA/pdf/16896102.pdf).
25. Nishiura H, Yanai H, Yoshiyama T, Kakehashi M: **Simple approximate backcalculation method applied to estimate HIV prevalence in Japan.** *Jpn. J. Infect. Dis.* 2004, **57**: 133-135 (Available at: http://www.nih.go.jp/JJID/57/133.html).
26. Hashimoto S, Fukutomi K, Morio S, Ichikawa S, Yamamoto N, Naemura M, Soda K: **Prediction of future trends of HIV infection and AIDS in Japan.** *Nippon. Koshu. Eisei. Zasshi.* 1993, **40**: 926-933 (in Japanese with English abstract).

27. Hashimoto S, Fukutomi K, Ichikawa S, Morio S, Niimura K, Soda K: **Study on trends of reported numbers of HIV infected and AIDS cases based on the AIDS surveillance data until 1994.** *Nippon. Koshu. Eisei. Zasshi.* 1995, **42**: 1091-1096 (in Japanese).
28. Hashimoto S, Fukutomi K, Ichikawa S, Matsuyama Y, Nakamura Y, Kihara M: **Future prediction of the number of HIV-infected persons and AIDS cases.** *J. AIDS. Res.* 2000, **2**: 35-42 (in Japanese with English abstract. Available at: http://api-net.jfap.or.jp/siryou/kenkyu/kihara/pdf/C071_S13.pdf).
29. Hashimoto S, Fukutomi K, Yamaguchi T, Matsuyama Y, Nakamura Y, Kimura H, Ichikawa S, Kihara M: **Attempt at long-term prediction of the numbers of HIV-infected persons and AIDS cases in Japan using system analysis.** *J. AIDS. Res.* 2002, **4**: 8-16 (in Japanese with English abstract. Available at: http://jaids.umin.ac.jp/journal/2002/20020401/200204010816.pdf).
30. Matsuyama Y, Hashimoto S, Ichikawa S, Nakamura Y, Kidokoro T, Umeda T, Kamakura M, Kimura S, Fukutomi K, Ikeda C, Kihara M: **Trends in HIV and AIDS based on HIV/AIDS surveillance data in Japan.** *Int. J. Epidemiol.* 1999, **28**: 1149-1155.
31. Centers for Disease Control and Prevention (CDC): *HIV/AIDS Surveillance Report, Vol. 15*. Atlanta: CDC; 2004.
32. Centers for Disease Control and Prevention (CDC): *HIV/AIDS Surveillance Report, Vol. 13 (No. 2)*. Atlanta: CDC; 2001.
33. Hashimoto S, Kawado M, Murakami Y, Ichikawa S, Kimura H, Nakamura Y, Kihara M, Fukutomi K: **Numbers of people with HIV/AIDS reported and not reported to surveillance in Japan.** *J. Epidemiol.* 2004, **14**: 182-186 (Available at: http://www.jstage.jst.go.jp/article/jea/14/6/182/_pdf).

Tables

Table 1 - Annual AIDS incidence in the United States from 1981-2003 and the predicted number of cases obtained based on a normally distributed epidemic curve

Year of diagnosis	Observed no. of cases†	Predicted no. of Cases‡	First ratio	Second ratio
1981	323	-	-	-
1982	1170	-	3.6223	-
1983	3076	-	2.6291	0.7258
1984	6247	-	2.0309	0.7725
1985	11794	-	1.8879	0.9296
1986	19064	-	1.6164	0.8562
1987	28599	-	1.5002	0.9281
1988	35508	37098	1.2972	0.8647‡
1989	42768	41612	1.1217	0.8647
1990	48732	40360	0.9699	0.8647
1991	59760	33850	0.8387	0.8647
1992	78705	24548	0.7252	0.8647
1993	78954	15394	0.6271	0.8647
1994	72266	8347	0.5422	0.8647
1995	69307	3914	0.4689	0.8647
1996	60613	1587	0.4054	0.8647
1997	49062	556	0.3506	0.8647
1998	41605	169	0.3032	0.8647
1999	41356	44	0.2621	0.8647
2000	41267	10	0.2267	0.8647
2001	40833	2	0.1960	0.8647
2002	41289	0	0.1695	0.8647
2003	43171	0	0.1465	0.8647
Total	915469	277765		

† Observed total number of AIDS cases in the United States. Data source: refs. [31,32].

‡Predicted number of cases based on observed cases from 1981 through 1987. The constant ratio, 0.8647, is equivalent to the value adopted in ref. [12].

Figures

Figure S1 - Compartments used to determine the rate of new HIV infection, diagnosis and progression to AIDS

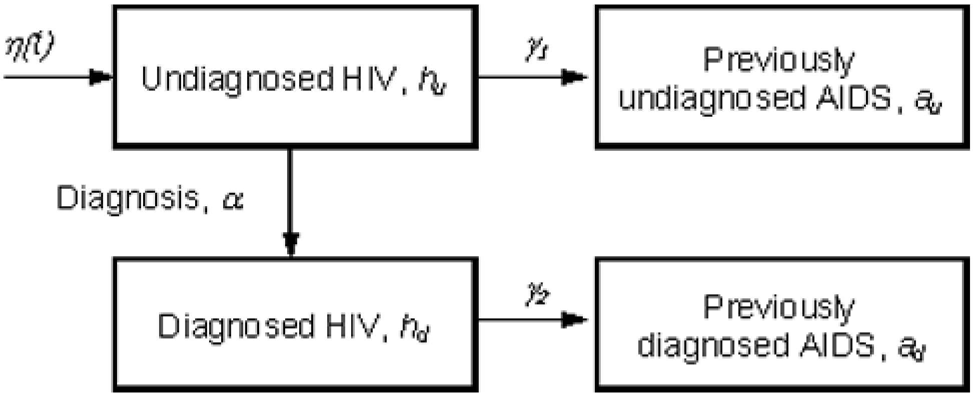

New HIV infection occurs as a function of time, $\eta(t)$. Undiagnosed HIV-infected individuals, h_u, develop into AIDS at a rate of γ_1 and are diagnosed at a rate of α (entering compartment h_d, diagnosed HIV infections). Diagnosed HIV individuals develop AIDS at a rate of γ_2. The coverage ratio employed in the Japan case studies was obtained from a_d/a_u.

Figure S2 - Comparative distributions of the coverage ratio of AIDS (a_d/a_u) and the ratio of diagnosed / undiagnosed HIV infections ($a_d + h_d / a_u + h_u$)

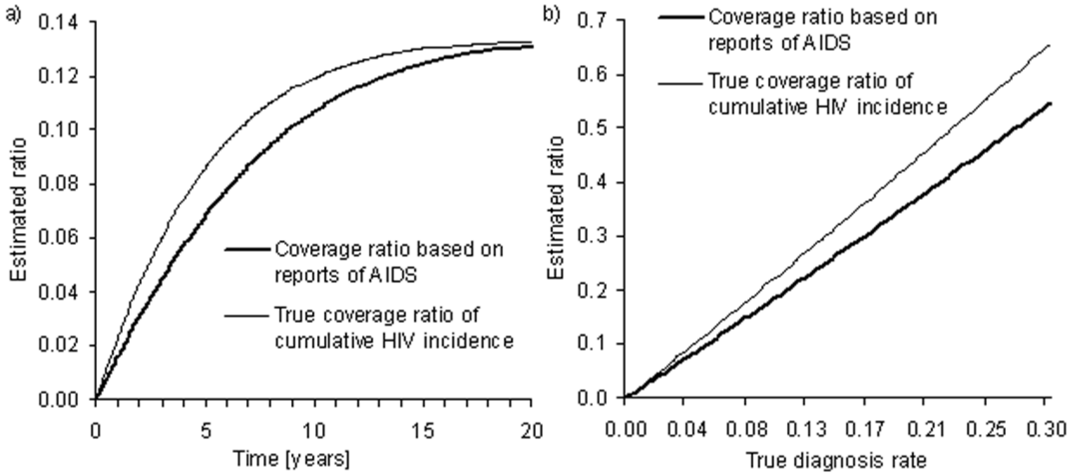

a) Temporal distributions of the coverage ratio and the ratio of diagnosed / undiagnosed HIV infections. The diagnosis rate, α, was fixed as 0.05 [year^{-1}] over time. The incubation period of AIDS was assumed to follow an exponential distribution with a mean of 10 [years] (= γ^{-1}). The values of η_0 and ρ were 111.0 and 0.273, as adopted from ref. [33]. **b**) Sensitivity of the estimated ratios (at time t = 7 [years] since the start of the epidemic) to the different true diagnosis rate, α. Other parameters were set as given in **a**.
